# Comparative analysis of biomechanical response between zygomatic implant and Facco technique through the three-dimensional finite element method

**DOI:** 10.4317/jced.59885

**Published:** 2023-02-01

**Authors:** Elton-Facco-Alves Costa, Marcela-Moreira Penteado, Bruno-Sotto Maior

**Affiliations:** 1 MSc and PhD in Dental Implantology from São Leopoldo Mandic University, Campinas, São Paulo, Brazil; 2 PhD in Prosthesis from Department of Dental Materials and Prosthodontics, Institute of Science and Technology, São Paulo State University, São José Dos Campos, São Paulo, Brazil; 3 Adjunt Professor Faculty of Dentistry of University Federal of Juiz de Fora

## Abstract

**Background:**

The placement of zygomatic implants is an alternative used for rehabilitation of edentulous patients with atrophic maxilla. However, the complexity of the various techniques suggested in the literature requires high skill from surgeons. Aim: The objective of this research was to compare the biomechanical performance of traditional technique of zygomatic implant placement in relation to a new proposal, the Facco technique, through finite element analysis.

**Material and Methods:**

A three-dimensional geometric model of the maxilla was input into computer-aided design software (Rhinoceros version 4.0 SR8). STL file of the geometric models of implants and components supplied by the company Implacil De Bortoli was converted to volumetric solids through reverse engineering by RhinoResurf software (Rhinoceros version 4.0 SR8). Three groups were modeled: traditional technique, Facco technique without frictional contact and Facco technique with frictional contact, following the recommended position in each technique for implant placement. All models received a maxillary bar. Groups were exported to the computer-aided engineering software ANYSYS 19.2, in step format. Mechanical static structural analysis was requested with occlusal load of 120N. All elements were considered isotropic, homogeneous, and linearly elastic. Contacts were considered ideal and system fixation was considered at the bone tissue base.

**Results:**

There is similarity between the techniques. Microdeformation values capable of generating undesirable bone resorption were not observed in both techniques. Highest values in the posterior region of Facco technique were computed at the angle of part B close to the posterior implant.

**Conclusions:**

Biomechanical behaviors of the two evaluated zygomatic implant techniques are similar. Prosthetic abutment (pilar Z) modifies the distribution of stresses over the zygomatic implant body. Highest stress peak was found in the pilar Z, but it is within acceptable physiological limits.

** Key words:**Atrophic maxilla, zygomatic implants, surgical techniques, pilar Z, dental implants.

## Introduction

Implant-supported complete dentures are increasingly used in the rehabilitation of edentulous patients. Thus, several protocols are studied in order to achieve success in the most diverse clinical cases. However, dental implants need adequate maxillary alveolar bone, limiting the treatment of patients who have presented the most severe form of alveolar resorption like maxillary resection by oncology in the region, congenital disorders or atrophic maxilla from early tooth loss (1).

In unfavorable situations for bone grafting techniques, maxillary sinus augmentation and Lefort I osteotomy with interpositional bone graft, one solution is zygomatic implants, proposed by Brånemark in 1989 (2). Since then, several authors have modified the original technique, which proposes the placement of an implant in the zygomatic bone on each side associated with at least two conventional implants in the anterior region (3,4). Preference for one modification technique over the other is related to anatomical conditions of the patient maxilla and zygomatic implants can provide shorter healing time, lower risks of contamination and consequently shorter treatment time (2). However, the degree of difficulty for technique execution makes them unattractive to the surgeon, since minor flaws may cause sinusitis, orbital cellulitis, subperiosteal fistulas, paresthesia, epistaxis, perforation of the orbital wall and infratemporal fossa (5,6).

Facco technique proposes a simpler way of placing implants in the zygomatic arch up to the alveolar crest, using a device composed of three parts of commercially pure grade IV titanium (Implacil de Bortoli, São Paulo, Brazil). Part A is a Morse taper implant with sandblasted surface with SLA acids. Part B is a smooth, polished, Z-angled pillar, with length of 18mm. At one extremity, it contains an internal Morse taper connection without indexing and at the other extremity, it contains a connection with a 12-mm internal thread. There is also a passant screw of 1.4-mm diameter. Part C is a 15-mm long rod, with a 10-mm thread at one extremity that connects to part B. It contains a self-threading nut for height delimitation and an external hexagon prosthetic platform with height of 0.7mm.

Implant design innovation in Facco technique promises a safer and less invasive surgical approach, as it is a totally extrasinusal technique, in addition to favoring direct vision for drilling and placing the zygomatic implant. Thus, it can even be performed in a dental clinic, avoiding the need for hospitalization, being a more attractive feature for patients. The objective of this research was to compare biomechanical behavior between the traditional technique of zygomatic implant placement and the Facco technique, through finite element analysis.

## Material and Methods

**Figure 1:**
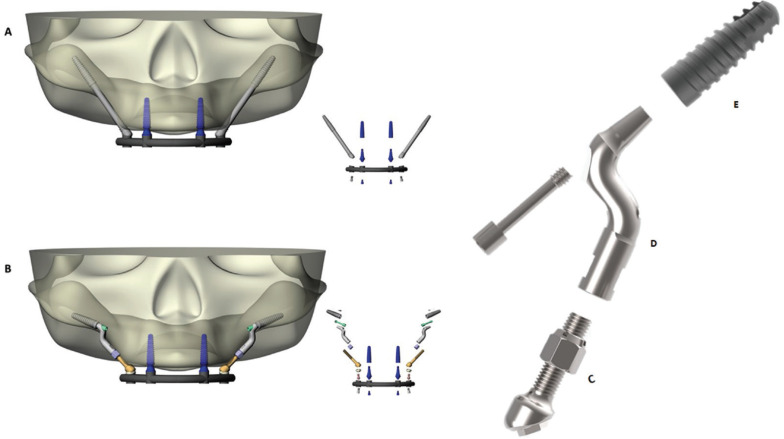
3D models for transpacency of tradicional zygomatic implant (A) and Facco´s techinique (B). Pilar Z prototype: Part C is a 15-mm long rod, with a 10-mm thread at one end that connects to part D. It contains a self-threading nut for height delimitation and an external hexagon prosthetic platform with a height of 0.7mm. Part D is a smooth, polished, Z-angled pillar, with length of 18mm. At one extremity, it contains an internal morse taper connection without indexing and at the other extremity, it contains a connection with a 12-mm internal thread. There is also a passant screw of 1.4-mm diameter. Part E is a morse taper implant with sandblasted surface with SLA acids.

**Figure 2:**
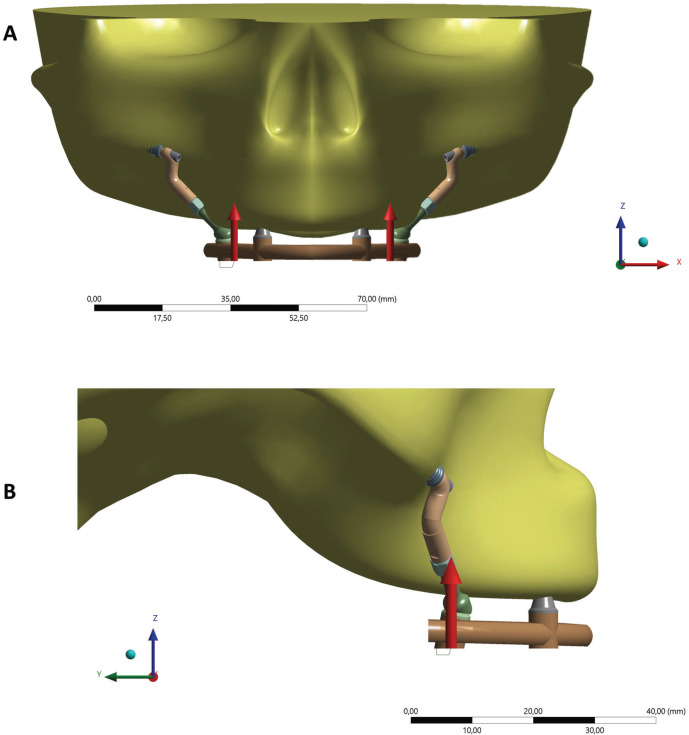
Frontal and lateral views of occlusal load simulated.

**Figure 3:**
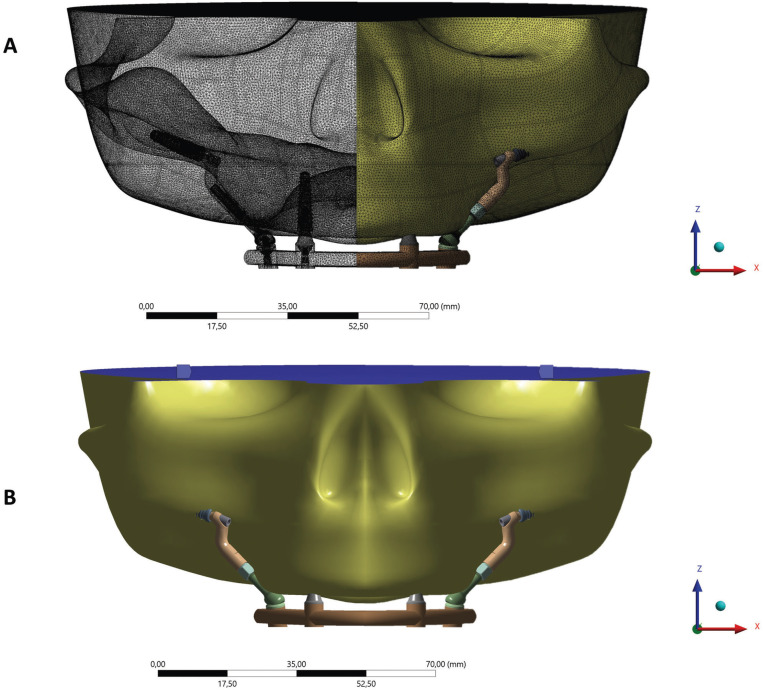
Mesh with tetrahedric elements (A) and fixation of system at base of model (B).

**Figure 4:**
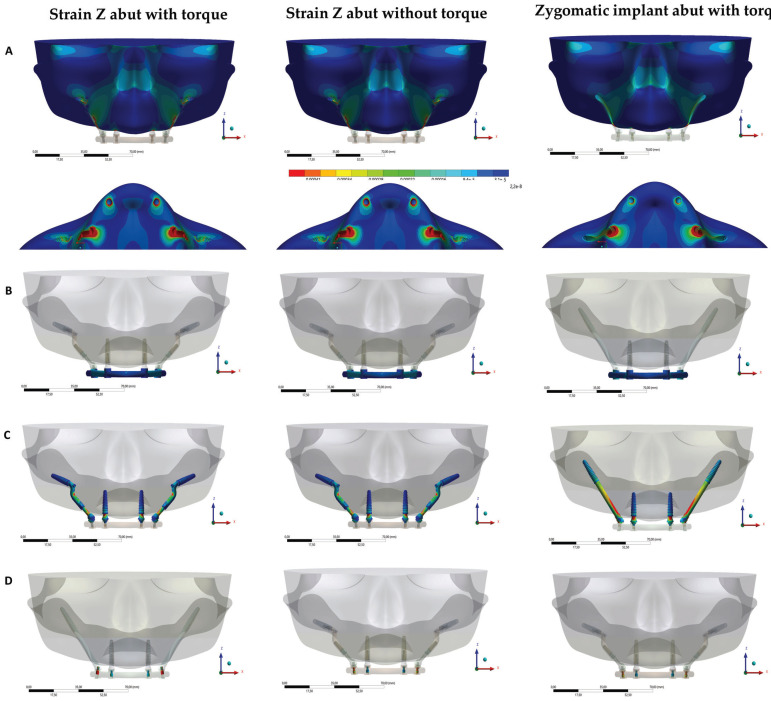
Bone microdeformation at vestibular and occlusal view (A) and Von-Mises tensile at bar (B), implants (C) and screw (D) with and without friccional contact.

For this study, a three-dimensional geometric model of the maxilla previously reported in the literature was selected (6). The bone tissue presented characteristics of periodontal health and absence of any anatomical changes (Figs. 1-4). Then, the model was input into computer-aided design software (Rhinoceros version 4.0 SR8; McNeel North America, Seattle, WA). Flat section cuts were used to isolate the lower portion of the skull. Verification of the absence of defective surfaces was performed manually through the analysis of the edges used in 3D modeling.

To obtain the geometric models of implants and components, STL files (stereolithography) were provided by the company (Implacil De Bortoli, Sao Paulo, Brazil). Conversion from STL to volumetric solid was performed by automated reverse engineering by RhinoResurf software (Rhinoceros version 4.0 SR8; McNeel North America, Seattle, WA). The set of component parts of Pilar Z were positioned according to clinical indication.

In the model with Facco technique, anterior implants (4.0x15 mm) were placed from the posterior region of the tuber to the canine and lateral region, measuring 45 mm, bilaterally. Then, conventional implants were inserted in the region of zygomatic arch 8 mm distant from the lower anterior border of zygomatic bone from the region of the maxillary lateral incisors. The set formed by parts of Pilar Z system was positioned according to implant prosthetic platform and according to the occlusal position of prosthetic screw access. For both anterior and posterior implants, mini conical abutments were modeled and positioned in the 3D model.

In addition to the experimental group, models of zygomatic implants were prepared for comparison. Zygomatic implants (40 x 4.3 mm) were developed containing 2.6 mm at the apex and 3.9 mm at the conical and threaded apical portion (ZAGA-Round, Straumann Dental Implant System, Waldenberg, Switzerland). Zygomatic implants were positioned according to the manufacturer’s instructions and according to Zygoma Anatomy Guided Approach (7).

For both models created, a maxillary bar was modeled containing dimensions of 4.5-mm height by 6-mm length and retained by 4 retaining screws.

Each finished model was exported to computer-aided engineering software (ANSYS 19.2; ANSYS Inc, Houston, TX) in STEP (Standard for the Exchange of Product Data) format for mesh division and finite element method analysis.

After importing the models, a mechanical static structural analysis was used to simulate the application of occlusal load (120 N). Then, the mechanical properties of each component used in the present study were defined based on the literature. The necessary properties were the elastic modulus and Poisson’s ratio of each material, assuming an isotropic, homogeneous, and linearly elastic mechanical behavior.

Then, contacts were manually defined between each structure, being considered ideal between structures. In maxillary region, two different contouring conditions were considered, one containing frictionless contact between the abutment and the surface of the vestibular cortical bone and another condition whose contact between these structures was frictional (Rough). Fixation of the system was defined at bone tissue base, in base region.

Subdivision of models into a finite number of nodes and elements was defined after the mesh convergence test with 10% linearity (8).

The required results were micro deformation in bone tissue (9) and von-Mises stress for implants and other metallic structures (10). In addition to the colorimetric stress distribution maps, the peaks of each analysis criterion were plotted for quantitative comparison.

## Results

There is a trend in the models for greater bone response in the posterior region than in the anterior region.

The analysis of the peri-implant tissue deformation evidences the comparison of models simulated by the applied force. In a study presented by Frost in 1994 (11), Wolff’s law and the behavior of bone structures in the face of different stimuli were reviewed. In this study, bone micro deformation values are assumed to be capable of modifying bone remodeling and apposition behavior, being used as safety parameters during the present simulation. Thus, values above 1500 με tend to activate lamellar bone remodeling, leading to reformulation and reinforcement, while values above 3000 με cause a remodeling disorganization that generates irreversible microdamage to the bone. Observing colorimetric graphs, it is possible to notice that micro deformation values capable of generating undesirable bone resorption were not calculated.

Observing the von-Mises stress maps, it is possible to notice that there is similarity between the Facco models with and without juxtaposition contact with the maxilla. However, model with zygomatic implants presents a different result of stress maps due to geometric alteration of implant system used. Regardless of the model, highest stress values in the anterior region were computed in the region of the emergence profile of the mini conical abutment. In the Pilar Z models, the highest values in posterior region were computed at part B angle close to posterior implant. For zygomatic implant, highest stress values were located in cervical region of the implant.

## Discussion

The use of alternative techniques allows the obtainment of specific results according to most convenient implant system with particularities of each clinical case (12). The present study created the frictional contact scenario to simulate the most hostile moment for performance of various implants (mastication).

 Results showed that Facco technique promotes a promising biomechanical response in dissipation of masticatory load independent of the contact with bone surface, since the Pilar Z provides new regions of stress concentration, with a lower value in the body of posterior implants in relation to traditional zygomatic implant.

The most critical region of Facco technique was the angle of part B close to posterior implant, reaching a peak tension of 28Mpa, that is, incapable of causing damage to the titanium or maxillary bone structure (Table 1). This finding corroborates Akay *et al*., 2015 (13), who observed three models of implants in zygomatics in a simulation of finite element analysis and concluded that maximum principal stress did not exceed the physiological limits of the maxillary bone.

**Table 1:**
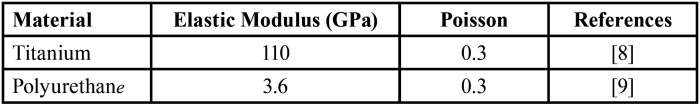
Mechanical properties.

Regarding the biomechanical behavior of the bar, stress distribution was similar, demonstrating that the emergence profile of anterior abutment and the cervical region of zygomatic implant (traditional or Pilar Z) support greater tension and prevent this load from being transmitted to the bar. This phenomenon corroborates the findings of Mousa *et al*., 2021 (14), who, after performing a systematic review of biomechanical stress in obturator prostheses using finite element analysis, concluded that the use of zygomatic implants reduced the displacement of prostheses.

Regarding retention screws, there was no statistically significant difference in all simulations. The biomechanical behavior of both techniques was observed to be similar, in this way, it is possible to assume that Facco technique may be a good option to introduce zygomatic implants with more facilities. However, all research protocols, including finite element analyses, have methodological limitations, so that computational numerical studies do not replace clinical studies. Finite Element Analysis (FEA) is a widely used numerical analysis that has been successfully applied in many areas of engineering and bioengineering. This computational numerical analysis may be considered the most comprehensive method currently available to calculate stress distributions in complex conditions by providing information that cannot be obtained through *in vitro* or even clinical studies (15).

Limitations inherent to the present method, however, do not allow the direct clinical extrapolation of the results without performing randomized clinical trials previously. Limitations include the use of materials with a homogeneous structure and isotropic behavior. There is no mismatch between components and surface defects. In addition, the implants were assumed to be 100% osseointegrated, although histomorphometric studies indicated that there is no 100% bone-implant interface.

## Conclusions

With the limitations of this research, it may be concluded that:

1) The biomechanical behaviors of two zygomatic implant techniques evaluated are similar.

2) The highest stress peak in Pilar Z is within acceptable physiological limits for stress dissipation in maxillary bone.

3) In terms of biomechanical behavior, Facco technique may be a viable alternative for zygomatic implant placement.

4) Randomized clinical trials are needed to validate the applicability of this innovation.
